# The role of visualization and 3-D printing in biological data mining

**DOI:** 10.1186/s13040-015-0056-2

**Published:** 2015-08-05

**Authors:** Talia L. Weiss, Amanda Zieselman, Douglas P. Hill, Solomon G. Diamond, Li Shen, Andrew J. Saykin, Jason H. Moore, for the Alzheimer’s Disease Neuroimaging Initiative

**Affiliations:** 1 Department of Genetics, Institute for Quantitative Biomedical Sciences, Geisel School of Medicine, Dartmouth College, Hanover, NH 03755 USA; 2 Division of Informatics, Department of Biostatistics and Epidemiology, Institute for Biomedical Informatics, Perelman School of Medicine, University of Pennsylvania, Philadelphia, PA 19104-6021 USA; 3 Thayer School of Engineering at Dartmouth, Hanover, NH 03755 USA; 4 Center for Neuroimaging and Indiana Alzheimer’s Disease Center, Department of Radiology and Imaging Sciences, Indiana University School of Medicine, Indianapolis, IN 46202 USA

## Abstract

**Background:**

Biological data mining is a powerful tool that can provide a wealth of information about patterns of genetic and genomic biomarkers of health and disease. A potential disadvantage of data mining is volume and complexity of the results that can often be overwhelming. It is our working hypothesis that visualization methods can greatly enhance our ability to make sense of data mining results. More specifically, we propose that 3-D printing has an important role to play as a visualization technology in biological data mining. We provide here a brief review of 3-D printing along with a case study to illustrate how it might be used in a research setting.

**Results:**

We present as a case study a genetic interaction network associated with grey matter density, an endophenotype for late onset Alzheimer’s disease, as a physical model constructed with a 3-D printer. The synergy or interaction effects of multiple genetic variants were represented through a color gradient of the physical connections between nodes. The digital gene-gene interaction network was then 3-D printed to generate a physical network model.

**Conclusions:**

The physical 3-D gene-gene interaction network provided an easily manipulated, intuitive and creative way to visualize the synergistic relationships between the genetic variants and grey matter density in patients with late onset Alzheimer’s disease. We discuss the advantages and disadvantages of this novel method of biological data mining visualization.

## Introduction

Biological data mining is inherently computational requiring artificial intelligence, machine learning, and/or pattern recognition algorithms to identify complex signals buried in noisy high-dimensional data. Much of the focus of research in these areas is on the development of more powerful algorithms that can solve data mining problems with minimal computing resources. Much less effort is focused on the interpretation of data models once discovered. Visualization has an important role to play in this process by allowing the researcher to see the data being analyzed and the research results that are generated. This can greatly facilitate the discovery process. Visualization is becoming more mainstream thanks to emerging technology such as 3-D televisions, virtual reality, and human-computer interactions tools such as touch computing. The purpose of this review is to introduce 3-D printing as one such technology. We briefly explore and illustrate this new technology using a case study from human genetics.

3-D printing is the process by which a physical object is manufactured directly from a digital model [1]. This is achieved through slicing the virtual model into a series of digital cross-sections and subsequently printing the real object layer by layer. Through this additive process, the physical object slowly gains volume until development is complete and the product is fully formed. 3-D printing techniques may differ slightly in aspects such as layering method and materials in accordance with the printer type and technology selected to match the requirements of final product or project. 3-D printing technology has applications in a variety of production and manufacturing industries including architecture, biotech, industrial design and engineering, but an area of interest that remains largely unexplored is the use of 3-D printing technology to study theoretical or nonphysical concepts. In this review, we explore the advantages and disadvantages of creating physical representations of intangible ideas. What value, if any, can 3-D printing bring to the scientific world of 3-D conceptual visualization?

## Case study from human genetics

Late onset Alzheimer’s disease, or LOAD, is an irreversible and progressive brain disease characterized by the development of neurofibrillary tangles and amyloid plaques and eventual death of neurons in individuals over the age of 60 and that symptomatically manifests as a progressive decline in memory, thinking, and reasoning skills [2]. The causes of LOAD are complex and not yet fully understood, but there is evidence that genetics plays a strong role in Alzheimer’s susceptibility and development. Genes that have been previously associated with Alzheimer’s disease include B-amyloid precursor protein, presenilin, presenilin 2, as well as apolipoprotein E. A database of genetic associations for Alzheimer’s disease exists [3] along with a resource provided by the National Institutes of Health for replicated genetic associations from genome-wide association studies of a variety of diseases including LOAD [4]. Despite significant effort, much of the heritability of LOAD remains unexplained.

One possible explanation for our inability to identify most of the genetic risk factors for LOAD may lie in the definition of the phenotype. The Alzheimer’s Disease Neuroimaging Initiative (ADNI) attempts to address this by tracing normal, mildly cognitively impaired, and Alzheimer’s disease brain changes to measure disease progression through utilization of MRI and PET imaging and laboratory and cognitive testing of over 800 patients in its first phase [5]. An important goal of this study is to identify new genetic risk factors for LOAD by using measures of brain structure and function as endophenotypes. These new brain imaging phenotypes may reveal additional risk factors that are not detectable using the higher level and noisier LOAD definition [6]. In addition, it is likely that genetic variants have synergistic interaction effects on LOAD risk that are not predicted from the independent marginal effects that have been identified thus far [7]. We summarize here previous studies of estimating gene-gene interactions and then present visualization of those patterns of association as networks. We then present a 3-D printed version of the network and discuss its usefulness.

### Construction of a gene-gene interaction network

A previous study by Zieselman et al. [8] described a gene-gene interaction network for grey matter density in Alzheimer’s patients from ADNI. The ADNI study measured approximately 500,000 single nucleotide polymorphisms (SNPs) [9]. Each pair of SNPs was assessed for its combined effect using the quantitative multifactor dimensionality reduction (QMDR) approach [10]. The final subset of statistically significant SNPs (n = 34) was selected and their genes assessed for biological interaction using the Integrative Multi-species Prediction (IMP) algorithm that integrates genomics data from thousands of sources [11]. The 34 statistically significant SNPs were used to build a statistical epistasis network as has been described previously [12]. As a first step toward printing the network, we developed a 3-D network visualization protocol (SNPAttractor) using the Unity 3D video game engine. This approach allow for the real-time visualization of genetic networks through the use of a gravitational model in which different parameters such as bond strength, number of positive and negative bonds, and node diameter can be changed and the effects seen in real-time. SNPAttractor allows for a digital 3-D representation of the structure along with the ability to explore the network in space by rotating around, moving through, and zooming in on the network. The SNPAttractor software and source code developed in Unity 3-D is available upon request.

### 3-D printing of a gene-gene interaction network

**Fig. 1:**
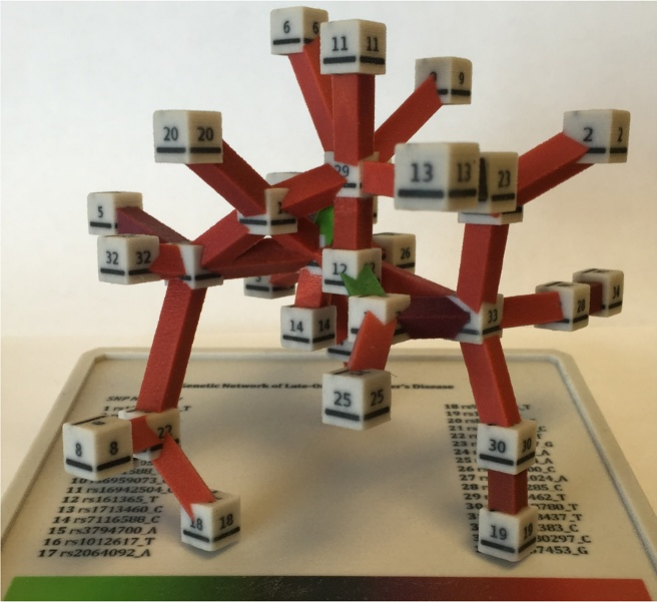
A genetic interaction network of Alzheimer’s disease as well as its base which contains SNP name labels and color key. The green edges in the network indicate stronger synergistic interactions

The first step in 3-D printing an object from a visualization is to convert the graphics file to the appropriate format that can be read by the printer. This is not always straightforward and we encountered some technical issues. First, the original SNPAttractor software doubled the face of intersection between the cylindrical connections and the nodes, resulting in files that were uninterruptable by the 3-D printer. Adjustments were made to the SNPAttractor code, and the edited files were uploaded successfully into the 3-D printer programs ZEdit and ZPrint and used to print a physical gene-gene interaction network using the ZPrint650 printer from 3D Systems, Inc. The process took the 3-D printer 10 hours. The final physical product can be seen in Fig. 1. The 3-D printed nodes are white cubes as opposed to the digital network’s black spherical nodes, and the printed connections are rectangular instead of cylindrical. The coloring of the connections on the digital versus physical model are identical, however, as the color represents the spectrum of possible node synergies (SNP interactions), ranging from the strongest synergy, represented by the color green, to the weakest synergy, represented by red. The 3-D printed network is roughly 12x12 cm, although the spokes provide an additional centimeter or two depending on orientation. It is important to note that no special support structures were needed for printing this network due the inherent strength of the printing material that was used. Indeed, it is no common to print objects using strong plastics and even metals. Because the SNP name could not fit on the surface of the nodes, each node was labeled with a number that corresponds with the SNP rs number. The number is printed on each face of the cube, so that the network can have multiple correct orientations and can be viewed from any angle. In addition to printing the genetic network, a base was printed to function as both a resting area for the structure and a key, where node number may be matched with SNP name and the synergy color scale may be referenced. The network is very light, slightly rough to touch, and can be picked up and handled with ease. It can be placed back on its base in numerous sturdy positions.

## Discussion

A single idea can be expressed in unlimited ways. Because of this, the field of data representation and visualization is in a continuous state of flux and evolution. The scientific community in particular is constantly trying to find new, creative ways to more easily and accessibly organize and interpret scientific data. A data set may remain unchanged, but the number of ways in which data can be displayed, viewed, represented, and subsequently interpreted are virtually limitless. It is through the application of this multimodal analysis process that we are able to gain a well-rounded understanding of the information that we wish to understand.

In this review, we used SNP biomarker data for Alzheimer’s disease to construct a three-dimensional digital representation of the gene-gene interaction network. We then created a physical model of the network using a 3-D printer to explore the advantages of using one or both of these visualization mediums. We suggest that useful information may be lost in the translation from physical structures to digital representations, and therefore propose that the use of a corporeal gene-gene interaction network model to supplement the digital SNPAttractor visualization software may inspire additional insights into the meaning and interpretation of the genetic network. In addition to the kind of biological or statistical networks presented in our case study, there are numerous other potential uses of this technology in biological data mining. For example, it might be helpful to print actual models. One could imagine printing a decision tree model derived from a source of big data. It might be interesting to print a phylogenetic tree or an ontology. An interesting challenge would be to print information visualizations such as scatterplots, barplots, boxplots, or even heat maps.

3-D visualization software and use of our digital genetic network offers many benefits but lacks intuitiveness and may therefore withhold important and possibly idea-stimulating information. We suggest that there are differences in the ability to intuitively recognize, understand, and subsequently interpret digital versus physical 3-D information. As physical beings in a three-dimensional world, we have evolved to expertly interpret our physical surroundings. Therefore, recognizing and understanding physical objects is, to a certain degree, intuitive. A problem arises, however, when we are asked to understand a digital representation of a physical object. Until recently, with the invention of computers and iPads, there have been no evolutionary pressures to hone our ability to interpret 3-D information through 2-D mediums, and therefore, such interpretation is unintuitive. A study by Lowrie [13] exposes the unintuitive nature of interpreting simulated 3-D objects. Lowrie investigated the ability of children to interpret screen-based images on the computer and relate them to real-world environments. Of 6 children, only 2 were able to find relationships between simulated 3-D and real-world 3-D environments. Lowrie goes further to suggest that the ability to infer relationships between simulated 3-D and actual 3-D environments can be enhanced through the construction and manipulation of 3-D models, a finding that demonstrates both the more innate nature of handling physical objects as well as the value of supplementing digital information with a physical counterpart. Other institutions reflect these ideas as well. For example, Kawakami [14] claims that because of the size and complexity of his molecular structures, digital model generation is difficult and peer discussions are laborious. In answer to this issue, Kawakami developed a physical, interactive protein model using 3-D printing technology that allows users to see, touch, and test ideas more easily and can be used in conjunction with digital applications. These examples highlight the additional intuitive benefit of supplementing digital visualizations with physical models.

How have we evolved to expertly interpret physical stimuli, and how are these modes of stimulus sensation and perception altered when we translate a physical object into the digital realm? Quite simply, we have evolved to sense and perceive real-world stimuli through five sensory modalities – sight, smell, taste, touch, and hearing. By translating a physical structure into the digital realm, we instantly eliminate the option to utilize four of these five senses. The efficacy of the remaining sensory modality – vision – is additionally drastically reduced during this translation. Visual resolution of the surrounding three-dimensional world is achieved through both stereopsis, the fusion of binocular images derived from retinal disparities to accurately communicate depth information [15], and monocular information, a more general but less accurate visual-perceptual method [16]. Although both of our digital and physical genetic network models are determined to be “three dimensional,” the difference resides in the method of presentation. While the physical network inhabits our tangible world, the digital network is presented through a 2-D medium – the computer screen. Therefore, interpretation of the former permits the use of stereopsis while interpretation of the latter is reliant on monocular cues, suggesting that we may lose valuable information in the translation from interpretation of physical to digital 3-D data. With a data set where complex relationships are expressed in 3-D space, the ability to accurately interpret these relationships is vital. We therefore suggest that 3-D information may be more accurately perceived through the handling and examination of a physical structure as compared to its digital counterpart.

Digital visualization provides many capabilities that physical models cannot, such as the ability to view various spatial arrangements and consequences of parameter change in real-time. However, we suggest that there may be advantages unique to experiencing this data through a physical medium that should not be ignored. Interpretation of a 3-D data set is both more intuitive and more accurate when experienced in the physical world as compared to the digital realm. Additionally, handling a physical model naturally stimulates discussion in group settings, allowing for new theories and ideas to be born. We therefore suggest that by supplementing our digital visualization techniques with a physical, tangible counterpart produced by 3-D printing technology, we may unlock ideas and insights about the data previously unattainable with only a digital model. Future studies should explore concept interpretation and comprehension in educational environments with use of digital visualizations with and without a supplementary physical counterpart.

In addition to these possible advantages of 3-D printing data objects it is also important to discuss some of the limitations of this technology. First, 3-D printing creates a static object that may not accurately represent the dynamics inherent in biological data. Once the object is printed it is fixed in time and space with one set of colors and shapes. In this sense, the visual display offered by a computer may be advantageous for many types of data and research results. It is worth noting that this disadvantage may be partially addressed by new 4-D technology that is able to print dynamic objects using thermal hydrogels [17]. Second, 3-D printing is likely to have size constraints. For example, it is unlikely that the typical hairball that is characteristic of large complex biological networks will be amenable to 3-D printing at the level of detail that is necessary to handle and interpret the object. Finally, it will be important to compare the usefulness of 3-D printed objects to other emerging technologies such as holograms that could be interacted with through haptic devices. It is our hope that this review will motivate formal scientific studies to evaluate the usefulness of 3-D printing and some of the other technologies mentioned for augmenting biological data mining.

## Availability

The software and source code for generating 3-D networks and 3-D printed material is available upon request.
